# Synergistic Antibacterial Activity and Wound Healing Properties of Selenium-Chitosan-Mupirocin Nanohybrid System: An *in Vivo* Study on Rat Diabetic *Staphylococcus aureus* Wound Infection Model

**DOI:** 10.1038/s41598-020-59510-5

**Published:** 2020-02-18

**Authors:** Reza Golmohammadi, Shahin Najar-Peerayeh, Tahereh Tohidi Moghadam, Seyed Mohammad Javad Hosseini

**Affiliations:** 1 Department of Bacteriology, Faculty of Medical Sciences, Tarbiat Modares University, Tehran, Iran; 2 Department of Nanobiotechnology, Faculty of Biological Sciences, Tarbiat Modares University, Tehran, Iran; 3 Molecular Biology Research Center, Systems Biology and Poisonings Institute, Baqiyatallah University of Medical Sciences, Tehran, Iran

**Keywords:** Cell biology, Microbiology, Diseases, Medical research, Pathogenesis

## Abstract

The current study aimed to formulate Selenium-Chitosan-Mupirocin (M-SeNPs-CCH) complex. The nanohybrid system was prepared using chitosan-cetyltrimethylammonium bromide (CTAB)-based hydrogel (CCH) that entrapped mupirocin (M) and selenium nanoparticles (SeNPs). The *in vitro* studies were performed by evaluation of the antibacterial activity and toxicity on L929 mouse fibroblast cell line. The *in vivo* study was conducted on rat diabetic wound infection model that was infected by mupirocin-methicillin-resistant *Staphylococcus aureus* (MMRSA). The wounds were treated by M-SeNPs-CCH nanohybrid system with concentrations of M; 20 mg/ml, CCH; 2 mg/ml and SeNPs; 512 μg/ml in two times/day for 21 days. The therapeutic effect of this nanohybrid system was evaluated by monitoring wound contraction and histopathological changes. Evaluation of the average wound healing time showed a significant difference between the treatment and control groups (*P*≤0.05). The histopathological study indicated that the amount of wound healing was considerable in M-SeNPs-CCH nanohybrid system groups compared to the control and M groups. The M-SeNPs-CCH nanohybrid system formulated in this study was able to reduce 3-fold MIC of mupirocin with synergistic antibacterial activity as well as to play a significant role in wound contraction, angiogenesis, fibroblastosis, collagenesis, proliferation of hair follicle, and epidermis growth compared to the control group (*P* ≤ 0.05). This research suggests that this nanohybrid system might be a development for the treatment of diabetic wound infection at mild stage.

## Introduction

Management of the infectious diabetic foot ulcer remains a global health issue^1,2^. According to the World Health Organization (WHO), about 422 million people worldwide suffer from diabetes^2^. About 25% of diabetic patients experience diabetic foot wounds in their lifetime^3,4^, half of them suffer infection^3^. Diabetic wounds are classified into three groups, i.e. mild, moderate and severe^3^. To avoid amputation and reduce health care costs, it is essential to control the infection^5^.

Gram-positive bacteria such as *Staphylococcus aureus* and beta-hemolytic streptococci are common infectious agents in the mild stage of diabetic foot wounds^3,5^. Regarding the increasing incidence of antibiotic-resistant strains such as methicillin-resistant *S. aureus* (MRSA)^6,7^, the treatment of infection caused by this organism has become more important^3,4^. Mupirocin antibiotic is used for the treatment of secondary skin infections by *S. aureus* (especially MRSA strains) and *Streptococcus pyogenes*^8^. However, according to the recent reports, mupirocin resistant *S. aureus* is on the rise^8,9^. Therefore, development of alternative antimicrobial drugs or increasing the efficacy of mupirocin is necessary^4,10^.

Studies on metal nanoparticles and drug delivery systems have shown that some of these materials can reduce minimum inhibitory concentration (MIC), thus increase the effectiveness of antibiotics^11,12^. Some reports are available on the antimicrobial activity and wound healing properties of selenium nanoparticles (SeNPs)^13–16^ and chitosan-based hydrogel^17–19^, but their efficacy such as MIC reduction along with mupirocin has not been studied so far. Selenium is a microelement that plays an important role in the health of the human body^15,20^. Chitosan with known antimicrobial effect^17,18^ is a biocompatible and biodegradable polysaccharide that is produced by deacetylation of chitin^17,21^, showing promising applicability in future drug delivery systems^17,18^. It is postulated that based on the antibacterial activity and wound healing properties of chitosan and SeNPs, combination of these two components may induce a synergistic effect, and their use along with mupirocin may help to increase its efficacy by reducing MIC. Moreover, it seems that chemical modification of chitosan with cetyltrimethylammonium bromide (CTAB) in the nanohybrid system can improve the antimicrobial activity of chitosan^17^.

The current study aimed to evaluate the antibacterial activity of M-SeNPs-CCH nanohybrid system against mupirocin-methicillin-resistant *Staphylococcus aureus* (MMRSA) in *in vitro* and *in vivo* for the first time. SeNPs and chitosan-N-cetyl-N,N,N-CTAB-based Hydrogel (CCH) were synthesized, characterized and optimized. Mupirocin was then entrapped in the SeNPs-CCH complex to formulate Selenium-Chitosan-Mupirocin (M-SeNPs-CCH) as the nanohybrid system. The *in vitro* studies were performed by evaluation of the antibacterial activity by MIC and checkerboard assays, followed by evaluation of the toxicity on L929 mouse fibroblast cell line. The *in vivo* experiments included creation of experimental wounds in diabetic rats and infecting them with MMRSA, followed by treating wound infection by M-SeNPs-CCH nanohybrid system and its individual components to monitor wound contraction and histopathological changes.

## Material and Methods

### Selenium nanoparticles (SeNPs) synthesis

A stock of aqueous solution of 16 mM (4.208 mg/ml) sodium selenite pentahydrate (Na_2_SeO_3_.5H2O) and 80 mM (14.089 mg/ml) ascorbic acid (Merck, Germany) were prepared. 24 ml deionized water was added to 1 ml of Na_2_SeO_3_ stock solution^22^. Ascorbic acid stock solution (1 ml) and tween 20 (20 μl) were added dropwise to Na_2_SeO_3_ solution under magnetic stirring at 1200 rpm at room temperature^22,23^. After changing the solution color to the light orange at 40 min, the mixture was diluted to 25 ml with deionized water. The nanoparticles were characterized by UV-Vis Spectrophotometer (Lambda25, USA), Dynamic Light Scattering (DLS), Zeta Potential (Malvern Zeta sizer, UK) and Transmission Electron Microscopy (TEM; Zeiss - EM10C −80 KV, Germany). The nanoparticles were purified by centrifugation at 10,000 rpm for 10 min for use in *in vitro* and *in vivo* studies^24^.

Selenium content of the mixture was determined by Atomic Absorption Spectroscopy (AAS)^25^. ImageJ 1.52i software was used to calculate the average diameter of the nanoparticles observed in TEM micrographs.

### Chitosan-N-cetyl-N,N,N-trimethylammonium bromide-based hydrogel synthesis (CCH)

The hydrogel was synthetized based on a study previously described by de Oliveira Pedro R *et al*. In the present study CTAB was used instead of alkyltrimethylammonium bromide^17^. Deacetylated low molecular weight chitosan (1.5 g) (Sigma, Germany) was dissolved in 1% acetic acid solution in the final volume of 20 ml and pH was adjusted to 9.0 by adding NaOH (0.5 M) under magnetic stirring (1200 rpm). 20 ml N-cetyl-N,N,N-trimethylammonium bromide solution (1.0 g, 137.1 mmol) was added while stirring. The process was continued at 60 °C for 72 hours and pH was monitored during the reaction time. The mixture was then dialyzed (MWCO 12KD, Sigma, Germany) to remove the unreacted CTAB in the following steps: against water for 2 days, then aqueous NaOH (0.05 M) for 1 day, and finally water for 2 days. The product was lyophilized and then characterized by Brunauer–Emmett–Teller (BET) surface area and Scanning Electron Microscopy (SEM)^26^.

After completing the synthesis and characterization of SeNPs and CCH, mupirocin powder (Syngen Biotech Co, LTD, Taiwan) was dissolved in polyethylene glycol 400 (Scharlau, Spain) to entrap in the SeNPs-CCH, and formulate Selenium-Chitosan-Mupirocin (M-SeNPs-CCH) as the nanohybrid system.

### Bacterial isolate

Methicillin-mupirocin-resistant *Staphylococcus aureus* strain (gifted from Microbial Bank of the Bacteriology Laboratory of Tarbiat Modares University) was used for all experiments.

### Antimicrobial susceptibility testing

The antimicrobial susceptibility testing was performed by disc diffusion and minimum inhibitory concentration (MIC) by broth dilution^27^ method. Double checkerboard assay was used to determine M-CCH and M-SeNp fractional inhibitory concentration (FIC)^28,29^.

### Toxicity assay

Dulbecco's Modified Eagle Medium-F12 (DMEM-F12, Sigma-Aldrich, USA) in 5 ml, 1 ml (10%) fetal bovine serum (FBS, Sigma-Aldrich, USA) and 1% Penicillin-Streptomycin (Aria CELL; Gibco/Sigma Source, Iran) were mixed and transferred to T-25 cell culture flask. The number of 1×10^4^ cells were seeded to the flask and incubated at 37 °C in a humidified atmosphere of 5% CO_2_ up to 7 days. After the third passage, the cells were harvested by Trypsin-EDTA 0.25 (Aria CELL; Sigma Source, Iran)^30,31^. Toxicity assay of M, SeNPs, CCH, along with their dual and triple combinations were tested in four folds higher than MIC concentrations using MTT cell viability assay kit (DNA Biotech Co, Iran) on mouse fibroblast connective tissue cell lines (L929, Iran Biological Resource Center), according to the manufacturer's protocol. The cells of each well were incubated with 5 mg/ml of MTT solution for 4 hours. Afterwards, formazan crystals were dissolved by dimethyl sulfoxide (DMSO) solution (DNA Biotech Co, Iran). The absorbance was measured at 570 nm by ELISA reader (Labsystem MultisKan, USA), and the cell viability percentage was calculated according to company protocol (DNA Biotech Co, Iran).

#### *In vivo* study

Diabetes induction: Approval of Tarbiat Modares University Ethics Committee was obtained before beginning the *In vivo* study [IR.TMU.REC.1395.414]. We declare that all methods were performed in accordance with the relevant guidelines and regulations. A total of 30 adult male Wistar rats weighing 225 ± 15 gr were fed with a high-fat diet containing 25% lipids (10% cholesterol). Two weeks later, the single low-dose of streptozotocin (STZ; Sigma) at a concentration of 40 mg/kg body weight was injected intraperitoneally to induce type 2 diabetes^32,33^. The rats’ blood glucose levels were measured after 72 hour by blood glucose monitoring systems (Medisign, Korea).

Surgery, experimental infection and treatment: Three days after the induction of diabetes, the diabetic rats were anesthetized with 30 mg/kg ketamine (Alfasan, Holland) and 4 mg/kg xylazine (Alfasan, Holland). Their dorsal hair was shaved and disinfected with iodine, and full thickness round wound was created in the interscapular region of the upper back of each rat and the skin was excised using a punch biopsy (with a diameter of 8 mm) and iris scissors^34–36^. All of the wounds were inoculated with freshly 10^8^ CFU mupirocin-resistant MRSA strains (30 μl). Immediately, the Comfeel Transparent (Coloplast, Denmark) were placed on the wounds and dressed with bundling. Three days after inoculation, all the wounds infections were confirmed by observation of infectious secretions and the presence of bacteria (gram staining and cultivation)^35,36^. The infectious wounds were divided into five groups including group 1: control/not treated group (Infectious Wound: IW), group 2; M, group 3; M-CCH, group 4; M-SeNPs, and group 5; M-SeNPs-CCH. Depending on the treatment groups, the wounds were treated by mupirocin (20 mg/ml)^36^, CCH (2 mg/ml) and SeNPs (512 μg/ml) twice a day for 21 days. Four of 30 rats (13.3%) died during the period of induction of diabetes, surgery and treatment.

Macroscopic and microscopic evaluation: The images were taken from wounds on days 1, 3, 7, 10, 14, 17, and 21. The average area of wounds was measured by ImageJ 1.52i software. The percentage of wound contraction was calculated using the following equation:

$${\rm{Percentage}}\,{\rm{of}}\,{\rm{wound}}\,{\rm{contraction}}=\frac{{\rm{Initial}}\,{\rm{wound}}\,{\rm{size}}-{\rm{Specific}}\,{\rm{day}}\,{\rm{wound}}\,{\rm{size}}}{{\rm{Initial}}\,{\rm{wound}}\,{\rm{size}}}$$

Tissue sampling was carried out for each group on days 3, 7, 14, and 21. All tissue samples were placed in a 10% formalin solution for 24 hours, dehydrated, and paraffin embedded using a tissue processor (DS 2080\H; Did Sabz Co, Iran). The tissue sections were stained with hematoxylin and eosin (H & E) and Trichrome-Masson staining was used for histopathological studies. The angiogenesis, fibroblastosis, hair follicles proliferation, epidermis growth, inflammation and collagenesis were evaluated in all tissue sections^37–39^.

Statistical analysis: Data was analyzed by SPSS 16 software. Non-parametric Kruskal–Wallis test was used because of the low sample size in each group for comparing the target groups and Mann–Whitney test was used for comparing the groups. P-value less than 0.05 was considered as statistically significant.

## Results

### Characterization of SeNPs

**Figure 1:**
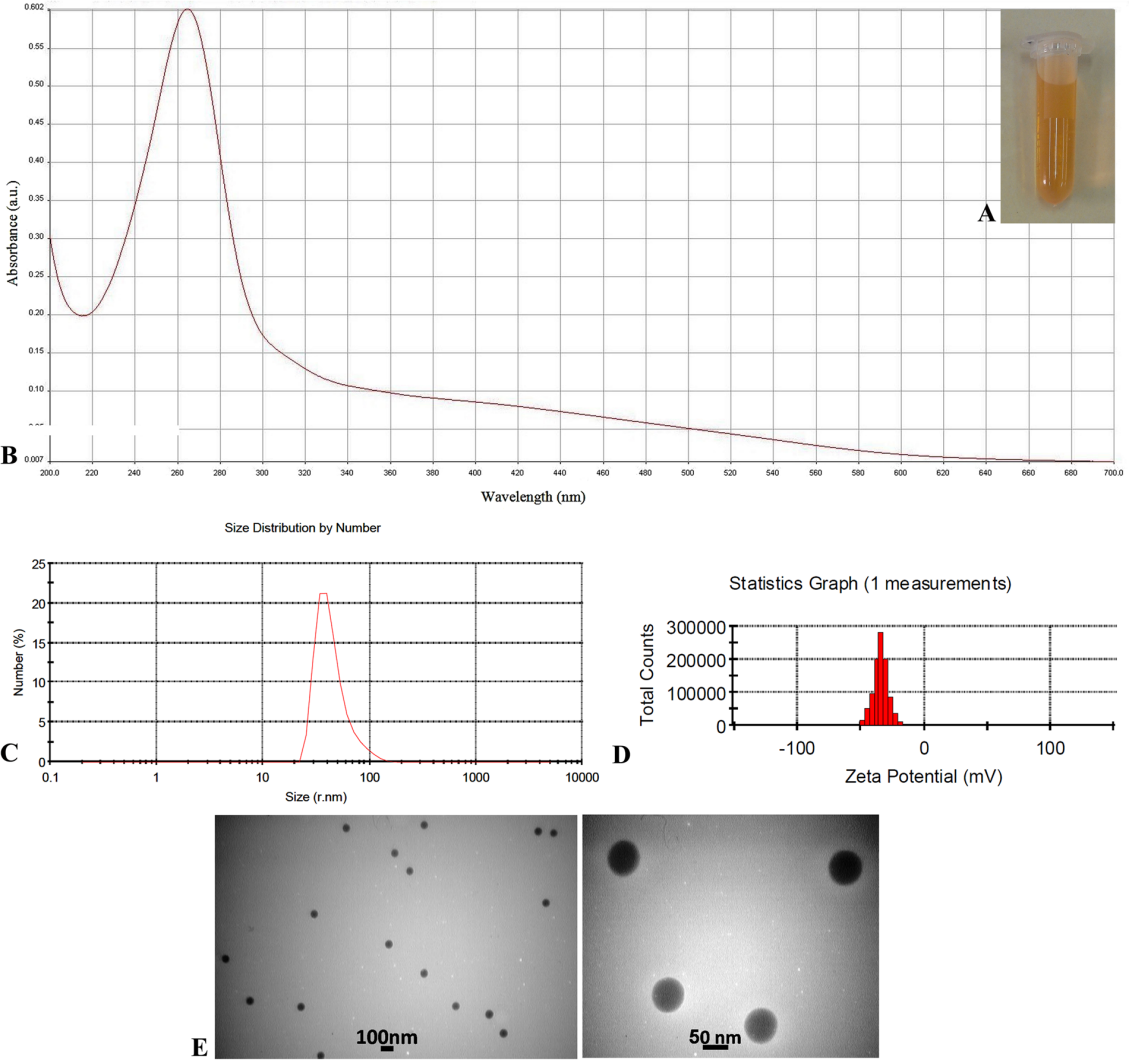
Characterization of selenium nanoparticle (SeNPs): (**A**) Appearance of the colloidal suspension, (**B**) UV-Vis spectrum, (**C**) Size distribution graph by number, (**D**) Zeta potential analysis, (**E**) Transmission Electron Microscopy images.

Appearance of SeNPs colloidal suspension and their characteristic UV absorption band are shown in Fig. 1(A,B). The PDI and Zeta potential of SeNPs were 0.170 and −34 mV, respectively (Fig. 1C,D). The mean diameters of SeNPs were measured 66 ± 8 and 61 ± 7 nm by DLS and TEM, respectively (Fig. 1C,E). A total of 75 nanoparticles in five TEM images were analyzed using ImageJ software.

### Characterization of CCH

**Figure 2:**
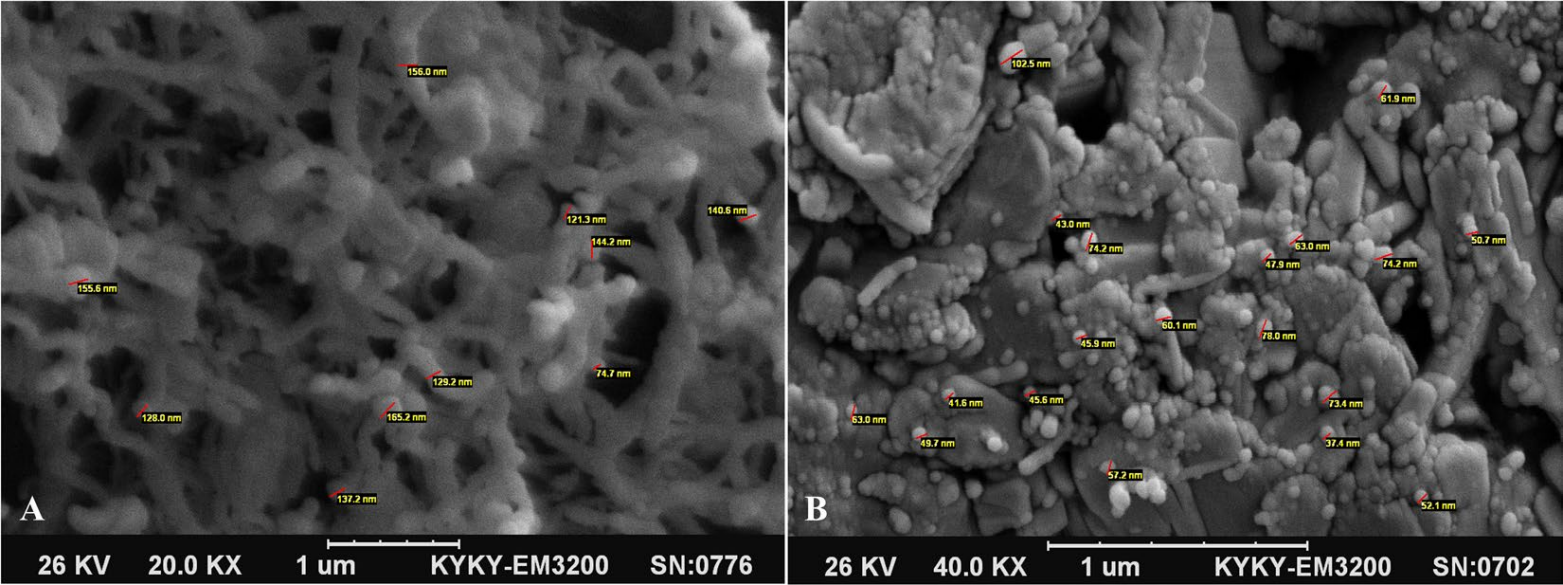
Scanning Electron Microscopy images of CCH and CCH loaded with SeNPs: (**A**) CCH; the red scales show the diameter of hydrogel fibers at nanometer scale. (**B**) CCH loaded with SeNPs; the red scales show the diameter of SeNPs loaded in CCH. CCH: chitosan-N-cetyl-N,N,N-trimethylammonium bromide-based hydrogel, SeNPs: selenium nanoparticles.

The BET and Langmuir surface area of CCH were measured to be 0.9680 and 2.3074 m^2^/g, respectively. The SEM images of CCH and SeNPs-loaded CCH are shown in Fig. 2. The mean diameter of SeNPs-loaded CCH was 59 ± 7 nm (Fig. 2B).

### Antimicrobial susceptibility testing results

The macrobroth dilution result showed that the MIC value of M, SeNPs and CCH against selected mupirocin resistant MRSA strain were 256, >256 and 256 μg/ml, respectively.

According to the checkerboard assay method, the FIC index value in the range of ≤0.5, 0.5–1, 1–4 and >4 are considered as synergistic, additive, indifferent and antagonistic effects, respectively. Based on the double checkerboard findings, the FIC index of M-CCH was 0.5 (at concentrations of 64 μg/ml for each of M and CCH) representing a synergistic effect that decreased 2-fold MIC of mupirocin. Moreover, based on this method, using 256 μg/ml SeNPs decreased 1-fold MIC of mupirocin (in the range of FIC 0.5–1, additive effect). The M-SeNPs-CCH (256 μg/ml for each substance) caused 3-fold reduction in the MIC value (32 μg/ml). The disc diffusion result of M, M-SeNPs, M-CCH, M-SeNPs-CCH (256 μg for each of substance) showed 19, 22, 23 and 24 mm for diameter of inhibition zone, respectively. In other words, the highest antibacterial activity belonged to the nanohybrid system, i.e. M-SeNPs-CCH.

### Toxicity assay results

**Figure 3:**
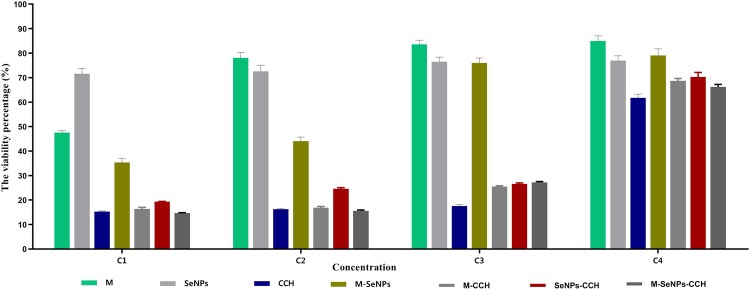
The viability percentage of L929 mouse fibroblast cell exposed to M, SeNPs, CCH, M-SeNPs, M-CCH, SeNPs-CCH and M-SeNPs-CCH for 24 hours at four concentrations below (C1 to C4): C1; M and CCH = 8192, SeNPs = 1024 μg/ml, C2; M and CCH = 4096, SeNPs = 512 μg/ml, C3; M and CCH = 2048, SeNPs = 256 μg/ml, C4; M and CCH = 1024, SeNPs = 128 μg/ml. The results were obtained based on the independent triple tests. M: mupirocin, SeNPs: selenium nanoparticles, CCH: Chitosan-N-cetyl-N,N,N-trimethylammonium bromide-based hydrogel.

Results of the toxicity assay revealed that the highest viability level at the same concentration (1024 μg/ml) was related to M (85%) in comparison with SeNPs (71.6%) and CCH (61.8%). The viability percentage of M, SeNPs, CCH, as well as their dual and triple combinations are shown in Fig. 3. The results were the same based on the independent third test (*P* ≤ 0.05).

#### *In vivo* study

**Figure 4:**
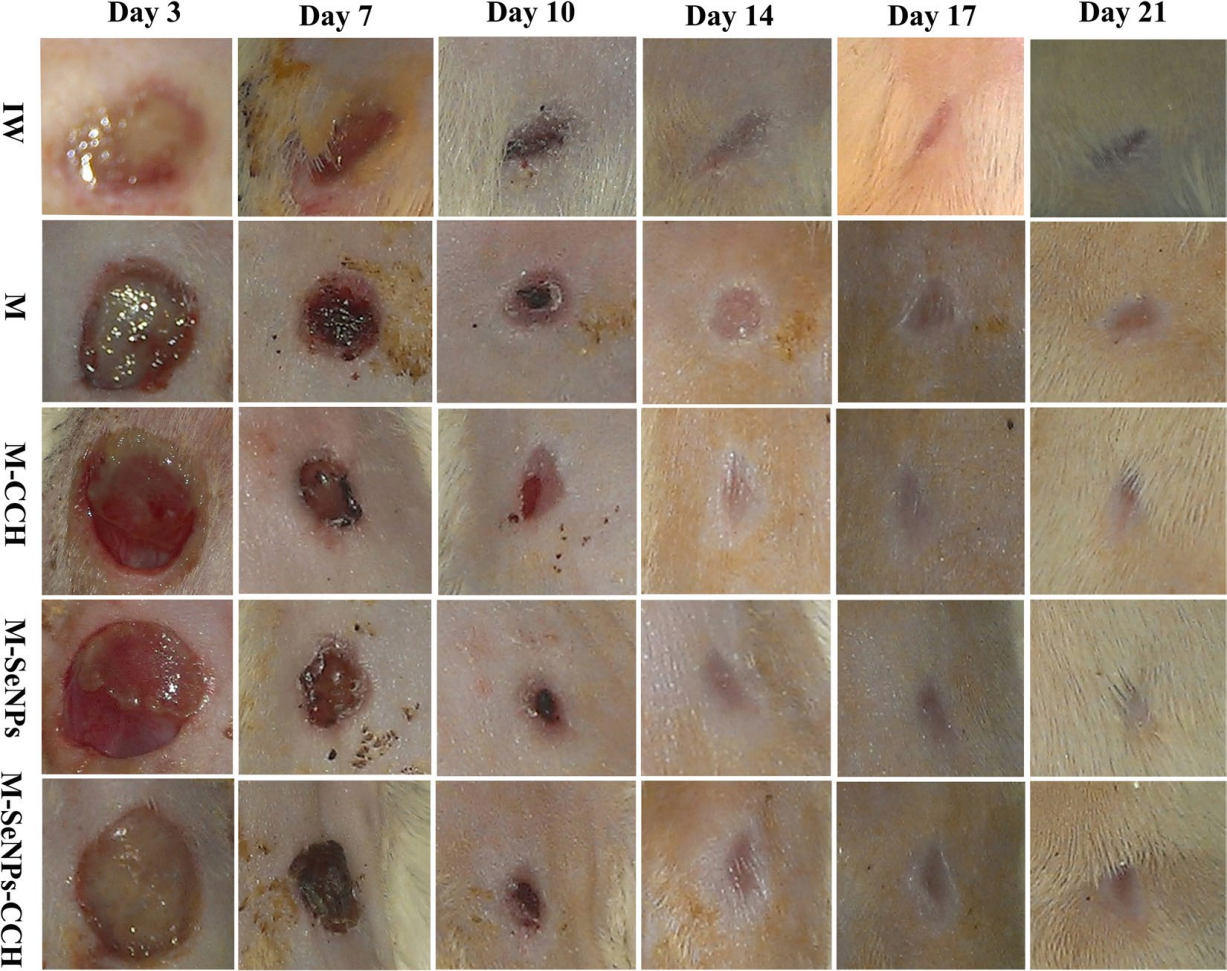
The wound healing images of untreated (IW) and treated groups (M, M-CCH, M-SeNPs, M-SeNPs-CCH) on days 3, 7, 10, 14, 17 and 21. The results were obtained based on triple independent tests (*P* ≤ 0.05). IW: infectious wound, M: mupirocin, SeNPs: selenium nanoparticles, CCH: chitosan-N-cetyl-N,N,N-trimethylammonium bromide-based hydrogel.

**Figure 5:**
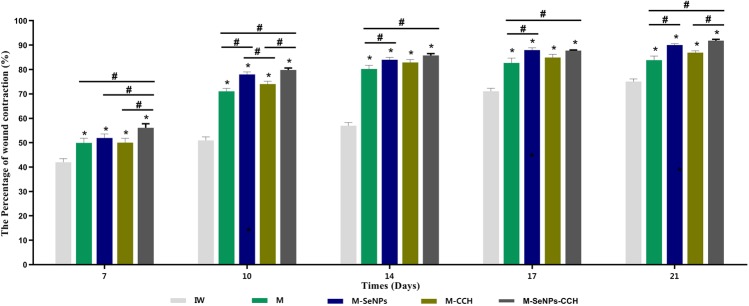
The percentage of wound contraction of untreated (IW) and treated groups (M, M-CCH, M-SeNPs, M-SeNPs-CCH) on days 7, 14 and 21. The calculation was performed by ImageJ software. The treating was started on the third day, but it is not included in the figure. *(*P* ≤ 0.05) shows significant differences between the treated and control groups and #(*P* ≤ 0.05) between the treated groups by Mann-Whitney test. The data were presented as mean ± SD. The results were obtained based on the triple independent tests (*P* ≤ 0.05). IW: infectious wound, M: mupirocin, SeNPs: selenium nanoparticles, CCH: Chitosan-N-Cetyl-N,N,N-trimethylammonium bromide-based hydrogel.

Macroscopic evaluation: The macroscopic evaluation results showed that the average wound healing of treated groups (M-SeNPs-CCH, M-SeNPs, M-CCH and M) were higher than infectious wound (IW) group on days 7, 10, 14, 17 and 21 (Fig. 4). The M-SeNPs-CCH-treated group showed considerable healing of 80% in 10 days. The M-SeNPs and M-SeNPs-CCH-treated groups showed 90 and 92% in 21 days, respectively. In addition, the wound contraction in M-SeNPs-CCH and M-SeNPs-treated groups was higher than M and M-CCH-treated groups. The percentage of wound healing is shown in Fig. 5 during 21 days. Measurement of the treatment size showed that M-SeNPs and M-SeNPs-CCH groups have higher wound contraction in comparison with other groups during 21 days. The highest percentage of wound healing belonged to M-SeNPs-CCH group with 92% in 21 days.

Based on the statistical analysis results, the average wound healing time of the treated groups (M-SeNPs-CCH, M-SeNPs, M-CCH and M) were significant compared to the IW group on days 7, 10, 14, 17, and 21. Also, the significant relationship between the treated groups on days 7, 10, 14, 17 and 21 were as follows: M-SeNPs to M on days 10, 14, 17 and 21; M-SeNPs-CCH to M on days 7, 10, 14, 17 and 21; M-SeNPs to M-CCH on day 10; M-SeNPs-CCH to M-CCH on days 7, 10 and 21; and M-SeNPs-CCH to M-SeNPs on day 7. The results were obtained based on three independent tests (*P* ≤ 0.05).

**Figure 6:**
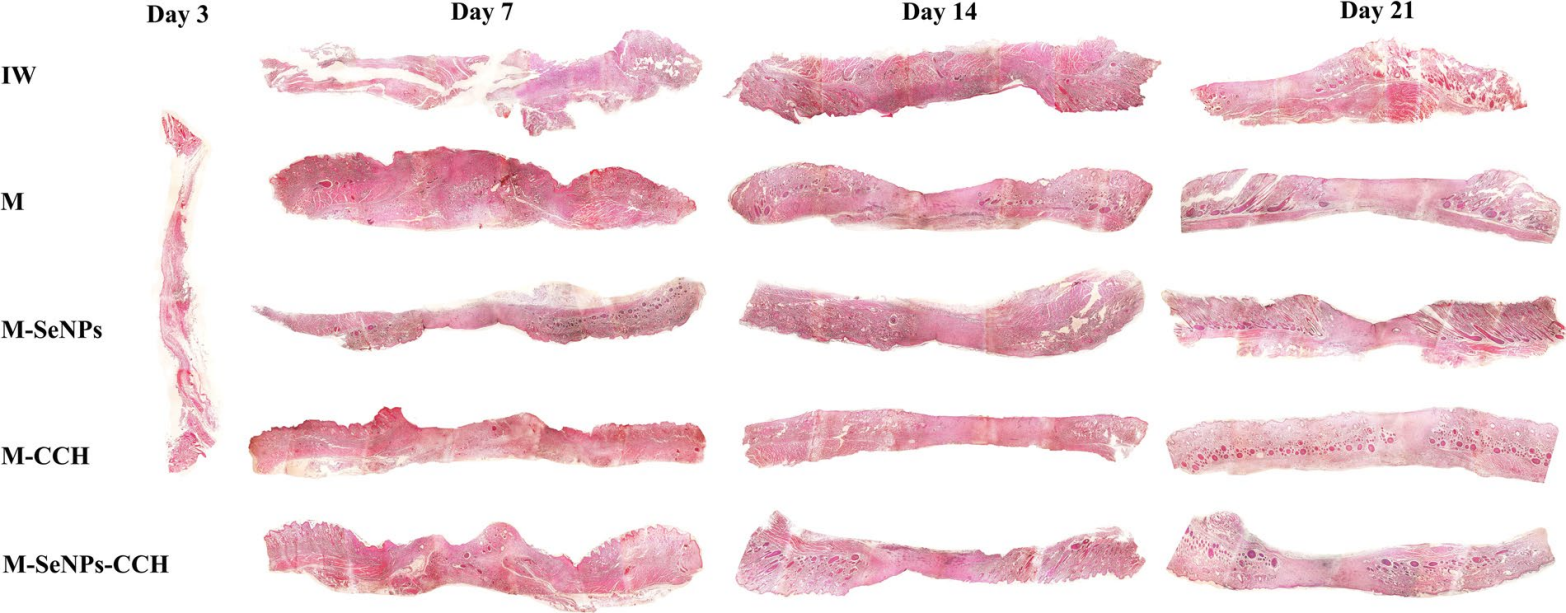
The H & E staining of tissue sections of wound groups on days 3, 7, 14 and 21 (magnification ×40). The cell count results obtained using these images are presented in Table 1. The results were obtained based on the independent triple tests. IW: infectious wound, M: mupirocin, SeNPs: selenium nanoparticle, CCH: chitosan-N-cetyl-N,N,N-trimethylammonium bromide-based hydrogel.

**Figure 7:**
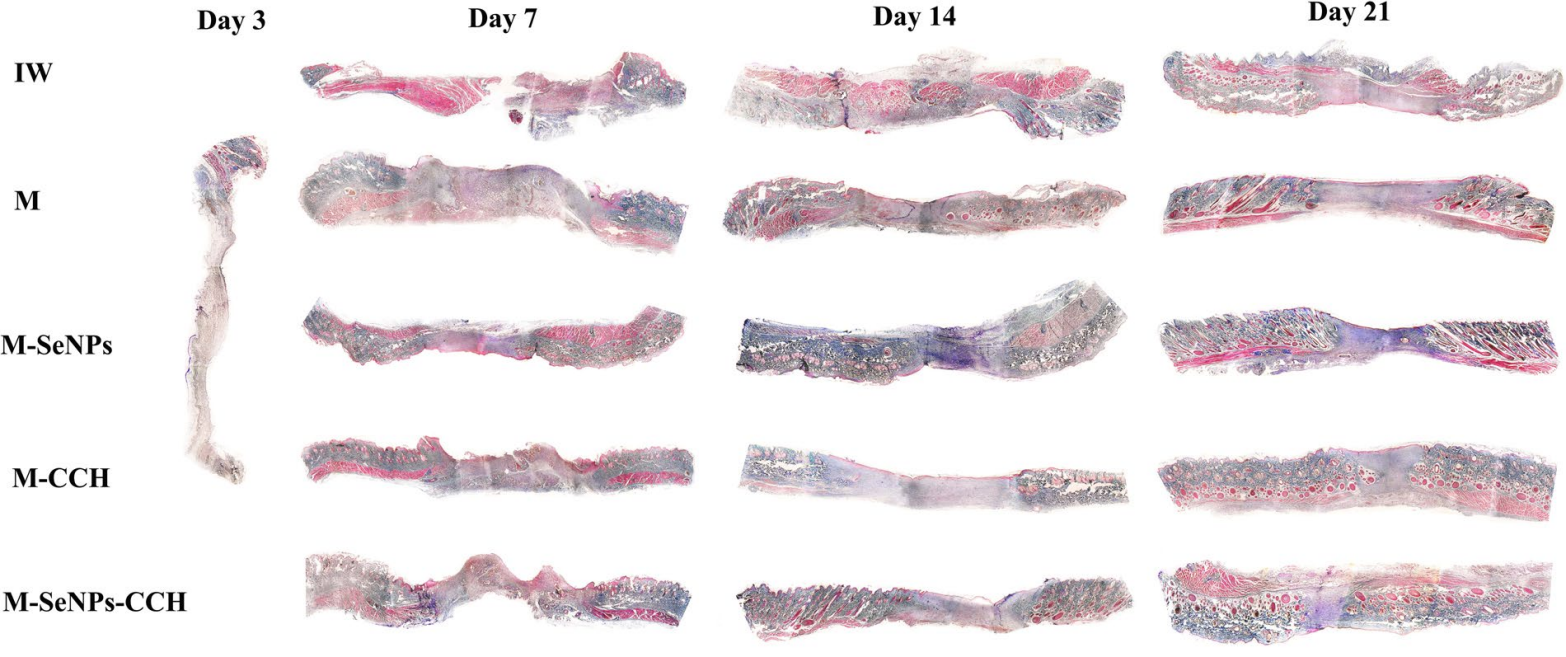
The Trichrome-Masson staining of tissue sections of wound groups on days 3, 7, 14 and 21 (magnification ×40). The cell count results obtained using these images are presented in Table 1. The results were obtained based on the independent triple tests. IW: infectious wound, M: mupirocin, SeNPs: selenium nanoparticles, CCH: chitosan-N-cetyl-N,N,N-trimethylammonium bromide-based hydrogel.

Microscopic evaluation. The results showed that three treated groups (M-SeNPs, M-CCH and M-SeNPs-CCH) had optimum wound healing in comparison with other groups. Wound healing increased gradually and reached the peak on day 21 compared to days 7, 10, 14 and 17. H & E and Trichrome-Masson staining showed that the amount of wound healing was considerable in M-SeNPs-CCH, M-SeNPs and M-CCH groups (Figs. 6, 7). The peak of vascularization was on day 7 which is related to M-CCH and M-SeNPs (5.1 and 3.7, respectively). Furthermore, these numbers were considerable in M-CCH, M-SeNPs and M-SeNPs-CCH groups on day 14 (4.1, 3 and 3.5, respectively). The number of fibroblasts increased in M-CCH, M-SeNPs and M-SeNPs-CCH groups on day 7 (13876, 8350 and 5510, respectively). This was also observed significantly in M-CCH, M-SeNPs groups on day 14 (26650 and 14430, respectively). In addition, the greatest growth and presence of collagen was noticed on days 14 and 21.These growths were 5.2, 4.6, and 2.2 in M-SeNPs, M-CCH and M-SeNPs-CCH groups on day 14, respectively. On day 21, the growths were 5.7, 3.7 and 4.9 in M-SeNPs, M-CCH and M-SeNPs-CCH groups, respectively. The amount of epidermis increased gradually during 21 days. Treated groups (M-SeNPs, M-CCH and M-SeNPs-CCH) had the maximum of the epidermis growth (136, 184.3 and 110, respectively) with the highest epidermis in M-CCH group on day 21. The amount of inflammation decreased during 21 days and the lowest rate belonged to M-SeNPs-CCH group. One of the quality criteria in wound healing is the growth of hair follicles. The results showed that the number of hair follicle increased in M-CCH group during 21 days (6 and 8 on days 14 and 21, respectively) (Table 1). The results were obtained based on three independent tests (*P* ≤ 0.05).

**Table 1:** The number evaluation angiogenesis, fibroblastosis, hair follicle and epidermis growth, collagenesis and inflammation in wound groups on days 3, 7, 14 and 21.

Days	Angiogenesis				Fibroblasts				Hair follicle				Epidermis				Collagen				Inflammation			
Groups	3	7	14	21	3	7	14	21	3	7	14	21	3	7	14	21	3	7	14	21	3	7	14	21
IW	0	1	2.2	3.9	2681	3387	6570	1050	0	0	0	0	0	0	12.1	39.9	0	0	1.2	2.2	0	1.4	1.2	1
M		2.4	1.5	1.4		3550	12400	7680		0	3	5		5.3	18.7	46.8		0	1.6	1.2		3.5*	2.2*	0.8*
M-SeNPs		3.7	3	1.3		8350	3840	3200		0	2	5		9.7	84.5	136		3.8*	5.2*	5.7*		1	0.5	0.5
M-CCH		5.1*	4.1*	1*		13876*	26650*	7785		0	6*	8*		35.6*	142.7*	184.3*		2.6	4.6	3.7		1.8	1.1	0.5
M-SeNPs-CCH		1.8	3.5	1.5		5510	14430	12500*		0	3	4		2.3	58.5	110		0	2.2	4.9		3.7	1	0.3

*Six tissue sections were evaluated for each sample at a set time and the statistical results showed that P-value less than 0.05 was regarded as statistically significant. IW: infectious wound, M: mupirocin, SeNPs: selenium nanoparticle, CCH: chitosan-N-cetyl-N,N,N-trimethylammonium bromide-based hydrogel.

## Discussion

In this study, SeNPs and CCH carrier systems were synthesized, characterized and optimized. Then, by entrapping mupirocin in the nanocarrier system, M-SeNPs-CCH was prepared. The SeNPs characterization result by UV/VIS spectrophotometer, DLS (PDI = 0.170, mean diameter = 66.48), zeta potential measurement (−34 mV) and TEM (mean diameter = 66.48) results indicated favorable characteristic features. BET and Langmuir surface area measurements of CCH were 0.9680 and 2.3074 m^2^/g, respectively. Taking the available reports on the antibacterial properties of individual selenium nanoparticles and chitosan into consideration, the M-SeNPs-CCH nanohybrid system in this study was designed to investigate its possible synergistic effects on mupirocin MIC reduction and wound healing in the rat diabetic infection model. To increase the antibacterial properties of chitosan, cetyltrimethylammonium bromide (CTAB) was used during the preparation of chitosan hydrogel. The study conducted by de Oliveira Pedro *et al*., reported that propyl and pentyl trimethylammonium bromides induce greater antimicrobial activities on chitosan^17,18^. In the current study, we used CTAB to increase the antimicrobial activity of chitosan.

There are some studies on chitosan hydrogel in relation to this study. Verma *et al*., reported antimicrobial and wound healing activity for sericin-chitosan-capped silver nanoparticle (S/C-SNP)-loaded hydrogel^19^. Masood *et al*., treated diabetic wounds with chitosan-PEG-Silver Nitrate-based hydrogel, and confirmed improvement in antibacterial activity and diabetic wound healing^40^. Xie *et al*., found that entrapment of silver nanoparticles into chitosan hydrogels could increase its antibacterial activity. In addition to wound healing properties of hydrogel, this study also showed that the hydrogel significantly improved the re-epithelialization and collagen deposition^41^. Another study by Kumar *et al*. on chitosan hydrogel/nano zinc oxide composite bandages (CZBs) indicated that combination of zinc oxide nanoparticles with chitosan hydrogel improved antibacterial activity and wound healing. Moreover, the nanocomposite accelerated re-epithelialization and collagen deposition. Kumar *et al*. also suggested application of this system in treatment of diabetic wounds^42^.

The M-SeNPs-CCH system formulated in this study was able to reduce mupirocin MIC 3-fold by macrobroth dilution. Furthermore, M-SeNPs and M-CCH reduced the MIC value by microbroth dilution checkerboard assay one and two fold, respectively. Also, the checkerboard results of M-CCH represented an additive effect. Due to the difficulty of aggregating nature of M-SeNPs-CCH, the triple checkerboard was not performed, and the disc diffusion test was used instead. Based on the latter assay, increase in the diameter of inhibition zone of M-SeNPs, M-CCH and M-SeNPs-CCH (22, 23 and 24 mm, respectively) compared to the M (19 mm), revealed an increase in antibacterial activity.

Some studies have reported that SeNPs inhibit *S. aureus* growth^43–45^. In this study, although the SeNPs reduced the growth of bacteria by one fold, there was no total inhibition of bacteria by this nanoparticle. Therefore, to obtain complete inhibition by this nanoparticle, further investigations are required. Considering other properties of SeNPs, including immunomodulatory^20^, anti-inflammatory^46^, and anti-oxidant^47^ properties, it seems that these nanoparticles can be promising candidates for combination with other antibiotics.

The toxicity assay result of this study revealed that the highest viability level at the same concentration (1024 μg/ml) is related to M (85%); in comparison with SeNPs (71.6%) and CCH (61.8%). According to the previous studies, toxicity of SeNPs is less than that of Se^15,20^. In this research, reduction in the viability percentage of CCH (71.6%) compared to chitosan can be due to the presence of CTAB.

According to the macroscopic evaluation results, the amount of wound healing is as follow: M-SeNPs-CCH> M-SeNPs> M-CCH> M> IW. This indicates that SeNPs has a more efficient effect on wound contraction than that of CCH. Also, the wound contraction in M-SeNPs-CCH, M-SeNPs and M-CCH was more than M group, with a remarkable healing effect compared to the control group (IW). The wound contraction was notable in the M-SeNPs-CCH group for 21 days. The average wound healing time of the treated groups were significant compared to the IW group on days 7, 10, 14, 17, and 21 (*P* ≤ 0.05).

The microscopic results of H & E and Trichrome-Masson staining showed increment in the amount of collagenization and epidermization in all three groups of M-SeNPs, M-CCH and M-SeNPs-CCH during 21 days. Based on angiogenesis results, the M-CCH group was significant compared to the M-SeNPs group in all days (*P* ≤ 0.05). Also, the M-SeNPs and M-CCH groups were highly significant compared to the other groups in all days (*P* ≤ 0.05). In fibroblastosis, epidermis growth and hair follicle proliferation investigations, the M-CCH group was highly significant compared to the other groups (*P* ≤ 0.05). In collagenesis study, the M-SeNPs group was highly significant, and in inflammation results, it was significant compared to the other groups (*P* ≤ 0.05).

The two groups of M-CCH and M-SeNPs-CCH showed the highest levels of fibroblasts and optimum vascularization on day 14, showing their significant effect on wound healing. On the other hand, the amount of inflammation decreased sharply during 21 days. Histopathology evaluation showed that the acceleration of wound healing in two groups of M-CCH and M-SeNPs-CCH were much higher than the other treatment groups during 21 days. This indicated that the presence of chitosan hydrogel in M-CCH and the SeNPs-chitosan hydrogel in the nanohybrid system played a significant role in controlling the infection and accelerating the wound healing as well as forming the main layers of the skin during 21 days.

An overall comparison of the macroscopic and microscopic evaluation results indicated that the presence of SeNPs had a significant role in wound contraction and collagenesis, and the presence of CCH was effective in angiogenesis, fibroblastosis and proliferation of hair follicle and epidermis (*P* ≤ 0.05). Considering the role of SeNPs and CCH in this study, these substances appear to be essential in such types of formulations.

### Conclusion

The M-SeNPs-CCH nanohybrid system formulated in this study was able to reduce 3-fold MIC of mupirocin. The system also played a significant role in wound contraction, angiogenesis, fibroblastosis, collagenesis, and proliferation of hair follicle and epidermis compared to the control groups (*P* ≤ 0.05). In addition to antimicrobial activity against MRSA strain and reduction of MIC value of mupirocin, the SeNPs and CCH could play a vital role in the wound healing process in rat model of diabetic wound infection. Thus, it seems that the presence of SeNPs and CCH to be essential in this formulated system. Therefore, this research suggests that SeNPs and CCH can be considered as promising candidates in developing of mupirucin-based drugs for the treatment of diabetic wound infection at the mild stage.

## Data Availability

The data sets and analysis of current study are available from the corresponding author upon reasonable request.
